# Cancer Advocacy in Africa: case studies of innovative practices

**DOI:** 10.1186/1750-9378-8-S1-S7

**Published:** 2013-07-15

**Authors:** Folakemi T Odedina, Belmira Rodrigues

**Affiliations:** 1 Pharmaceutical Outcomes and Policy, College of Pharmacy, University of Florida, Gainesville, FL, USA; 2 African Organisation for Research and Training in Cancer (AORTIC), Cape Town, South Africa

## Abstract

In this paper, we present six case studies describing innovative cancer advocacy programs in Africa. For each case study, an example of an advocacy activity, list of factors contributing to the success of the organization, and an example of an obstacle addressed by the organization are described.

## Introduction

In 2012, the Africa Oxford Cancer Foundation (AfrOx), African Organisation for Research and Training in Cancer (AORTIC), European Society for Medical Oncology (ESMO) and the Union for International Cancer Control (UICC) developed the 1^st ^***Africa Cancer Advocacy Toolkit***, which was released in September 2012.[1] The toolkit was developed as a resource to show how advocacy can be used to improve cancer care in Africa and includes how to: (i) develop an advocacy plan; (ii) work with governments; (iii) create awareness raising events and resources; and (iv) secure funding. In order to develop a comprehensive synopsis of innovative cancer advocacy programs throughout Africa, AORTIC conducted a survey of cancer organizations following the release of the toolkit. An open-ended survey form (see Table 1) was employed to collect the information needed to develop the advocacy case studies. Thirty-nine cancer advocacy organizations participated in the survey between July and August, 2012. This paper describes six of the case studies developed from the survey. For each case study, an example of an advocacy activity, list of factors contributing to the success of the organization, and an example of an obstacle addressed by the organization are described.

**Table 1:** Cancer advocacy case study form

Describe a political, education, fundraising, community outreach, research, or support advocacy that **you successfully worked on within the last year**, providing comprehensive details on key aims, methods, actors, timeframe, and outcomes. In addition, provide a **Photovoice narrative** about this advocacy activity. A Photovoice strategy is a way for people to represent their community activities. For the Photovoice narrative, provide at least one photograph (maximum of three photographs) and describe the elements of the photograph(s) and their meaning in relationship to cancer advocacy. Please describe the key ingredients that have made your organization and its advocacy work to be successful. What were some key problems or obstacles that you faced in your advocacy work? Please provide at least one example of how you overcame an obstacle in advocacy.

## Case Study 1. Advocacy report from Tanzania Tobacco Control Forum (TTCF), Tanzania

### Advocacy program

An important advocacy program for us is the campaign to Tanzania’s politicians and the community at large to foster the passage, adoption and enforcement of the World Health Organization (WHO) Framework Convention on Tobacco Control (FCTC) compliant legislation. Tanzania has a Tobacco Products (Regulation) Act, 2003 (TPRA, 2003) which needs revision after the country ratified the WHO FCTC in April 2007. Subsequently, a review process was set up to ensure that the TPRA 2003 conforms to the WHO FCTC requirements. The Tanzania Tobacco Control Forum (TTCF) participated in this review process, which was conducted under the auspices of the Tanzanian Ministry of Health and Social Welfare (MHSW). Unfortunately, the proposed bill addressing the modification of the TPRA 2003 has not been tabled to the Tanzanian parliament. Due to this setback, TTCF made the decision to gather more evidence-based information on tobacco farming to be able to effectively fight the tobacco industry and convince parliamentarians on the hazards of tobacco and the need for adopting an effective tobacco control law. TTCF carried out tobacco impact surveys in the two major tobacco growing areas in Tanzania, the Songea and Tabora Regions. The evidence gathered from the survey study was presented to Members of Parliament (MPs) from five parliamentary committees. The MPs took the evidence very seriously and advised tobacco control advocates to prepare the draft of an FCTC compliant bill and present it to them for tabling to parliament. The MPs also advised that the collected evidence should be presented at a larger audience of MPs in order to gain more support. With the support of the Legal Consortium of the Tobacco Free Kids Action Fund, TTCF has prepared the draft bill, which will be presented as a Private Members' Bill. It is expected that the Bill will be presented at the coming November parliamentary session. TTCF is also planning to hold a bigger sensitization seminar to all MPs at the coming parliamentary session to facilitate the adoption of the proposed bill. The passage of an effective legislation will protect Tanzanians from tobacco smoke and therefore enhance public health.

### Factors contributing to the success of the organization

Our advocacy activities have been successful due to a feasible strategic plan, committed members and effective lobbying strategies.

### Obstacles and solutions

Our primary challenge is the tobacco industry interference, which led to the delay of passage of the FCTC compliant bill. We are overcoming this challenge by targeting and lobbying parliamentary members who are pro-tobacco control.

## Case Study 2. Advocacy report from the Ethiopian Public Health Association, Ethiopia

### Advocacy program

One of the key contributing factors to the “health of a nation” is the capacity of a public health system to identify and respond in a timely and adequate fashion to situations that affect the health and well-being of its people. The active participation of professional associations such as the Ethiopian Public Health Association (EPHA) in the formulation of policy as well as in the design, implementation and monitoring/assessment of programs, are essential elements of a successful public health strategy.

The advocacy program of EPHA focuses on: (i) enhancing and reinforcing the role of civil society, and (ii) raising the level of awareness and understanding of the impact of public policy on human health and well-being. EPHA identifies and addresses local health-based needs, develops relevant activities in response to health needs, and advocates for appropriate public policies. EPHA represents an independent voice without the constraints of professional licensing, certification or self-interest.

### Factors contributing to the success of the organization

The key ingredients that have contributed to the success of EPHA are continuous assessment, comprehensive media intervention, discussions with policy makers and community leaders, and the use of forums to dialogue with stakeholders.

### Obstacles and solutions

At the time of this survey, Ethiopia is one of the few African countries that have not been ratified by the WHO FCTC. This is a major challenge for EPHA. To overcome this challenge, we are working closely with other civil societies and the government and optimistic that Ethiopia will be endorsed by the WHO FCTC soon.

## Case Study 3. Advocacy report from Sebeccly Cancer Care and Support Centre, Lagos, Nigeria

### Advocacy program

Every year, we organize a cancer workshop for the media professionals on issues they can disseminate to the public. We believe that the more people who are informed on cancer prevention and control, the more cancer prevention or cancer cures we can achieve. Health care professionals can inform a few hundred but journalists can reach millions of people. The cancer workshop brings the media professionals, health care professionals, cancer survivors and advocates together. The objectives are to:

1. Promote information exchange.

2. Educate journalists (broadcast and print) about breast cancer early detection, treatment and survivorship issues.

3. Increase the frequency of media reporting on breast health, such as breast self examination, clinical breast examination, and mammograms.

4. Secure the commitment of media professionals on breast and other cancer information dissemination.

5. Create awareness about breast cancer survivorship and support.

Although the cancer workshop is an annual workshop, various activities take place throughout the year. A small group of dedicated journalists were recruited and trained to lead other journalists on a series of continuous media activities focused on cancer. The workshop includes an award dedicated to excellent cancer journalism (electronic and print) to recognize the best reporter of the year on cancer awareness and advocacy (BRCA Award).

### Factors contributing to the success of the organization

Our organization has been successful due to the following factors:

• A passionate focus on helping breast cancer patients.

• A dedicated and action oriented workforce, primarily comprising people who have been affected by cancer.

• Existing strategic partnership.

• Branding of our activities.

• Internal fundraising, which sustain overheads and activities in the absence of sponsor donations.

• Creating innovative services that tackle problems.

• Perseverance and professionalism.

• Having fun while working hard.

### Obstacles and solutions

Geographical coverage is one of the primary obstacles for our organization. We are based in Lagos, the most populous state in Nigeria. It is a challenge to cover the entire state as each local government area is densely populated. In overcoming this, we are partnering with other organizations and the Lagos State Ministry of Health to cover the whole state through the local government areas.

## Case Study 4. Advocacy report from Crusaders Club Ministry, Sierra Leone

### Advocacy program

Our organization has successfully raised awareness about cancer in Sierra Leone by targeting students. Students are our primary focus for various reasons, including the fact that it reinforces cancer prevention at an early age and also encourages dialogues of students with their parents and other family members. Students are educated through oral presentations and the use of international cancer posters. An innovative approach that we employ is the use of drama with students as actors to educate other students. The outcome of our cancer education advocacy has been very good resulting in decrease in smoking, decrease in alcohol intake and increase in the consumption of fresh vegetables and fruits among our target population.

### Factors contributing to the success of the organization

The commitment, dedication and passion of our staff, volunteers, and student participants have contributed significantly to the success of our organization.

### Obstacles and solutions

The primary obstacle for our organization is lack of funding to support the activities of the organization. We have been able to overcome this problem by reaching out to international organizations for in-kind support such as educational materials and posters. We also explore and use opportunities for staff training by organizations in Africa such as AORTIC and international organizations.

## Case Study 5. Advocacy report from Uganda Women's Cancer Support Organisation (UWOCASO), Uganda

### Advocacy program

One of the innovative approaches by our organization is the use of research to understand the needs of our target community. For example, we conducted a qualitative research to understand the local knowledge, perception and health seeking behavior regarding cancer in Uganda. Funded by the UICC, this study formed the basis for the Uganda Women's Cancer Support Organisation (UWOCASO) and other stake holders’ cancer advocacy programs and activities. The main objective of the project was to generate qualitative data that will be used by UWOCASO and other stakeholders to:

1. Establish the factors that lead to poor cancer screening and early detection programs.

2. Develop a communication and advocacy strategy for UWOCASO and other stakeholders.

3. Design appropriate materials for cancer prevention and control.

4. Develop a documentary video based on the findings of the research

Based on this project, UWOCASO has focused on diverse advocacy programs. One such program is Support Advocacy, using the Reach to Recovery (RR) concept. RR is a non-medical program which provides breast cancer patients and their families with practical and emotional one-on-one support to cope with breast cancer diagnosis and everyday life challenges after diagnosis. RR is built on the premises that a woman who has lived through breast cancer and gives her time and experience to help others confronting the same experience is a valuable support. This is a continuous program whereby breast cancer survivors support cancer patients and survivors through peer counseling. In addition, the RR program provides necessary resources like prosthesis, bras, and wigs to patients.

Another important advocacy program by UWOCASO is the celebration of World Breast Cancer Awareness Month in October annually. In 2009 and 2010, UWOCASO organized charity walks in Uganda. The walk marked the climax for October awareness activities. In both events, over 500 people from various communities, organizations, and institutions participated in the walk. The major objectives of these walks were to:

1. Raise awareness about breast cancer and highlight the importance of early detection for breast and cervical cancers.

2. Conduct breast and cervical cancer screening using a mammogram van.

3. Enhance collaboration with key stakeholders and partners.

4. Solicit support from potential sponsors for UWOCASO programs.

5. Advocate breast and cervical cancer.

On Friday February 4, 2011, UWOCASO partnered with the Ministry of Health, WHO, PATH Uganda and Save A Woman Initiative (SAWI) to commemorate World Cancer Day. This was an important day for all stakeholders to share information about the burden, interventions and challenges of cancer prevention in Uganda. There was a strong urgency to discuss “the bottlenecks in cancer prevention in the country and the way forward”. The objectives outlined for the World Cancer Day Breakfast dialogue were to: (i) create awareness about cancer; (ii) mobilize allies for cancer prevention and management; (iii) position cancer as a problem in Uganda; and (iv) promote early health seeking behavior for cancer prevention in Uganda.

### Factors contributing to the success of the organization

The success of UWOCASO is based on several factors, including the partnership with other organizations, collaborating with the Ministry of Health, and an organized team.

### Perceived obstacles and solutions

The major obstacle for UWOCASO is inadequate funds. Similar to other African countries, the health priority in Uganda is communicable diseases such as HIV/AIDS, malaria and tuberculosis. Our organization has been successful in securing grant funding from an international organization, the UICC, to support our program.

## Case Study 6. Advocacy report from the Breast Cancer Foundation of Egypt (BCFE), Egypt

### Advocacy program

Late breast cancer diagnosis and advanced stage disease in Egypt have been attributed to lack of awareness, lack of routine breast cancer screening, cultural factors and social habits. To address these problems, the Breast Cancer Foundation of Egypt (BCFE) implemented a comprehensive breast cancer screening program in 2005. Recognizing that BCFE alone cannot meet the need for breast cancer screening and effectively reduce the mortality of breast cancer in Egypt, BCFE founded the ***Breast Cancer Health Care Institutions Capacity Building Program*** in partnership with the following stakeholders: the Ministry of Health Teaching Hospitals, cancer centers, Ministry of health family health centers and the Egypt National Cancer Institute.

The overall goal of the Breast Cancer Health Care Institutions Capacity Building Program is to increase the number of breast cancer prevention and early detection clinics in governmental health care institutions. To facilitate the achievement of this goal, our primary aims include:

1. To train health care professionals about breast cancer prevention, early detection and screening programs.

a. Lectures and workshops for doctors and nurses about what is breast cancer and the methods of early detection diagnosis and management.

b. Training doctors to conduct physical examination.

c. Training technicians to perform high quality mammograms.

d. Training nurses to conduct breast self examination and teach it to other women as a method of early detection.

e. Training secretaries how to welcome women and complete clients’ data survey.

f. Introduction of breast cancer nursing in the educational curriculum in Egyptian faculties of Nursing.

2. To educate community health advocates about breast cancer prevention and early detection. BCFE conducts a training program for health advocates on how to teach women to do breast self exam monthly. In addition, an educational video is played in the waiting areas of early detection clinics to teach women basic information about breast health and early detection.

3. To educate the public about breast cancer prevention and early detection. BCFE provides health care institutions with breast cancer educational materials for distribution to patients. BCFE also implements a media campaign using television, radio and newspapers to educate the public about the services offered at breast cancer clinics.

One of our main achievements is that breast cancer early detection clinics are now available to women in different governorates at an affordable price. BCFE has assisted in the empowerment of the governmental care facilities to adopt policies of disseminating breast cancer awareness through breast cancer early detection clinics.

### Factors contributing to the success of the organization

Our organization leads in breast cancer awareness across the country because we: (i) present a unique model in providing awareness, diagnostic and rehabilitation services for women regardless of social or economical background; (ii) empower survivors physically and psychologically; and (iii) strengthen capacity building for other health entities to launch breast cancer early detection programs.

### Perceived obstacles and solutions

We face several challenges that affect our advocacy activities. Some of these challenges are:

1. Changing the culture of silence and taboos surrounding breast cancer.

2. Increasing public knowledge and understanding of breast cancer.

3. Encouraging Egyptian women to screen for breast cancer.

4. Encouraging breast cancer survivors to share their stories and to get involved in the community.

5. Fundraising is difficult because most people prefer to give donations to community development or children related causes such as education, illiteracy, and human rights.

BCFE implemented several actions to break the silence of the fear of breast cancer amongst the community. These actions included public events to strengthen BCFE’s profile in the community and at the same time raise awareness about breast cancer. The events included different approaches such as a walk, run, race, golf, zumba, screenings and talk shows. All the actions included a media campaign and electronic marketing to reach women, men, youth and children. BCFE focused on all social levels and clusters in urban, rural and squatter areas.

## Conclusion

The case studies presented in this paper are examples of innovative advocacy practices in Africa. In November 2013, AORTIC will publish an electronic supplement showcasing cancer advocacy by different organizations in Africa. In addition, the 2^nd^ biennial ***International Workshop on Cancer Advocacy for African Countries (CAAC)*** will include an ***Advocacy Expo & Posters*** session, featuring presentations by cancer advocates within and outside Africa. The workshop will take place on November 21, 2013 during the 9^th^ AORTIC International Cancer Conference in Durban, South Africa**.**

## List of abbreviations used

AfrOx: Africa Oxford Cancer Foundation; AORTIC: African Organisation for Research and Training in Cancer; BCFE: Breast Cancer Foundation of Egypt; CAAC: Cancer Advocacy for African Countries; EPHA: Ethiopian Public Health Association; ESMO: European Society for Medical Oncology; MHSW: Ministry of Health and Social Welfare; NGOs: Non-Governmental Organizations; RR: Reach to Recovery; SAWI: Save A Woman Initiative; TTCF: Tanzania Tobacco Control Forum; UICC: Union for International Cancer Control; UWOCASO: Uganda Women's Cancer Support Organisation; WHO: World Health Organization; WHO FCTC: WHO Framework Convention on Tobacco Control; UWOCASO: Uganda Women's Cancer Support Organisation.

## Competing interests

The authors declare that they have no competing interests.

## Declarations

The authors declare that funding for publication of the article was funded by an award provided by the National Cancer Institute (Award Number R13CA162899).

This article has been published as part of *Infectious Agents and Cancer* Volume 8 Supplement 1, 2013: Selected articles from The International Workshop on Cancer Advocacy for African Countries (CAAC) 2011. The full contents of the supplement are available online at http://www.infectagentscancer.com/supplements/8/S1 The Supplement Editors declare they have no competing interests.
